# Supplementary material for: Risk factors for sacrococcygeal pilonidal sinus: a systematic review and meta-analysis supplemented by genetic causal assessment
Source: Front Surg. 2026 Jan 7;12:1718589. doi: 10.3389/fsurg.2025.1718589 (PMC12819706; doi:10.3389/fsurg.2025.1718589)
Supplement: Supplementary file 2 [file Datasheet2.zip › Supplementary Data 2/MR_pipeline_p5e-8/scatter_plots/ebi-a-GCST006867.finngen_R12_L12_PILONIDALCYST.pdf]

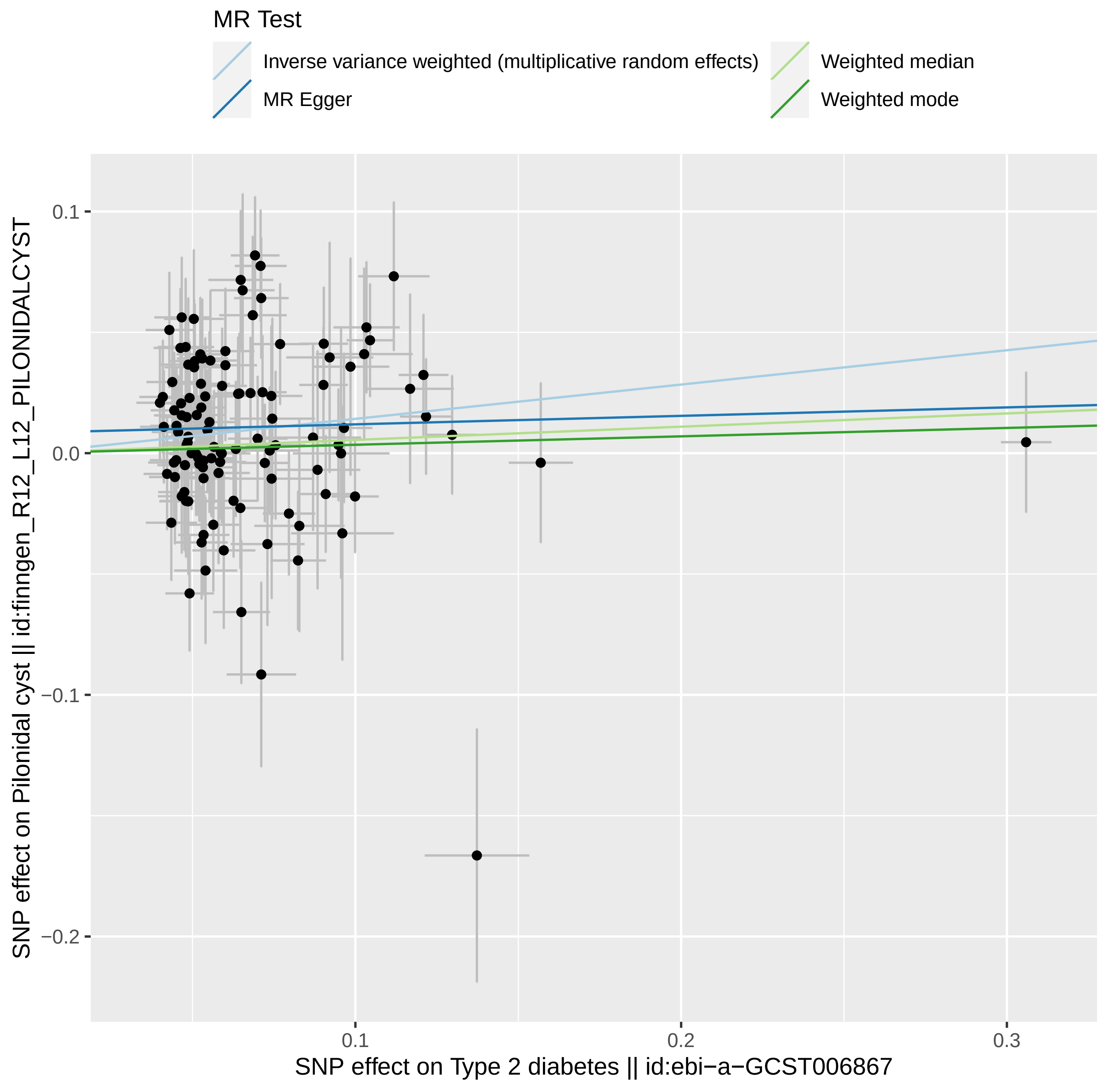

MR Test
Inverse variance weighted (multiplicative random effects)
MR Egger
Weighted median
Weighted mode
0.1
0.0
−0.1
−0.2
0.1
0.2
0.3
SNP effect on Pilonidal cyst || id:finngen_R12_L12_PILONIDALCYST
SNP effect on Type 2 diabetes || id:ebi−a−GCST006867
